# What are the main inefficiencies in trial conduct: a survey of UKCRC registered clinical trials units in the UK

**DOI:** 10.1186/s13063-017-2378-5

**Published:** 2018-01-08

**Authors:** Lelia Duley, Alexa Gillman, Marian Duggan, Stephanie Belson, Jill Knox, Alison McDonald, Charlotte Rawcliffe, Joanne Simon, Tim Sprosen, Jude Watson, Wendy Wood

**Affiliations:** 1 Nottingham Clinical Trials Unit, University of Nottingham, Nottingham, UK; 2 Clinical Trials and Statistics Unit, The Institute of Cancer Research, London, UK; 3 Cancer Research UK & UCL Cancer Trials Centre, London, UK; 4 Formally of Leicester Clinical Trials Unit, University of Leicester, Leicester, UK; 5 Barts Clinical Trials Unit, Queen Mary University of London, London, UK; 6 Centre for Healthcare Randomised Trials, University of Aberdeen, Aberdeen, UK; 7 Cancer Research UK, Liverpool Cancer Trials Unit, University of Liverpool, Liverpool, UK; 8 Formally of Bristol Randomised Trial Collaboration, University of Bristol, Bristol, UK; 9 Oxford Clinical Trial Service Unit & Epidemiological Studies Unit, University of Oxford, Oxford, UK; 10 York Trials Unit, University of York, York, UK; 11 NIHR RDS South Central, University of Southampton, Southampton, UK

**Keywords:** Randomised trials, Multicentre trials, Inefficiencies, Trial conduct, Survey, Clinical trials unit

## Abstract

**Background:**

The UK Clinical Research Collaboration (UKCRC) registered Clinical Trials Units (CTUs) Network aims to support high-quality, efficient and sustainable clinical trials research in the UK. To better understand the challenges in efficient trial conduct, and to help prioritise tackling these challenges, we surveyed CTU staff. The aim was to identify important inefficiencies during two key stages of the trial conduct life cycle: (i) from grant award to first participant, (ii) from first participant to reporting of final results.

**Methods:**

Respondents were asked to list their top three inefficiencies from grant award to recruitment of the first participant, and from recruitment of the first participant to publication of results. Free text space allowed respondents to explain why they thought these were important. The survey was constructed using SurveyMonkey and circulated to the 45 registered CTUs in May 2013. Respondents were asked to name their unit and job title, but were otherwise anonymous. Free-text responses were coded into broad categories.

**Results:**

There were 43 respondents from 25 CTUs. The top inefficiency between grant award and recruitment of first participant was reported as obtaining research and development (R&D) approvals by 23 respondents (53%), contracts by 22 (51%), and other approvals by 13 (30%). The top inefficiency from recruitment of first participant to publication of results was failure to meet recruitment targets, reported by 19 (44%) respondents. A common comment was that this reflected overoptimistic or inaccurate estimates of recruitment at site. Data management, including case report form design and delays in resolving data queries with sites, was reported as an important inefficiency by 11 (26%) respondents, and preparation and submission for publication by 9 (21%).

**Conclusions:**

Recommendations for improving the efficiency of trial conduct within the CTUs network include: further reducing unnecessary bureaucracy in approvals and contracting; improving training for site staff; realistic recruitment targets and appropriate feasibility; developing training across the network; improving the working relationships between chief investigators and units; encouraging funders to release sufficient funding to allow prompt recruitment of trial staff; and encouraging more research into how to improve the efficiency and quality of trial conduct.

## Background

Randomised trials are the gold standard for evaluating the effects of interventions to improve health and wellbeing. Trials addressing important health care questions are often large multicentre studies, which are complex, expensive multidisciplinary projects. Inefficiencies in the conduct of trials may lead to wasted resources, to an extension of the trial or, in extreme circumstances, to the trial failing to complete or to answer the research question [1, 2].

The UK Clinical Research Collaboration (UKCRC) registered Clinical Trials Units (CTUs) Network aims to support high-quality, efficient, effective and sustainable clinical trials research in the UK (http://www.ukcrc.org/research-infrastructure/clinical-trials-units/). Currently the network includes 50 CTUs, and these units primarily conduct multicentre randomised trials. Since 2012, the network has developed a work programme to support members with information, guidance and representation relevant to high-quality trial conduct. The Efficient Trial Conduct subgroup of this work programme aimed to explore new approaches and systems to improve trial conduct and share good practice. To better understand the challenges facing CTUs in conducting trials efficiently, and to help prioritise its work, the Efficient Trial Conduct subgroup surveyed staff working within the registered CTUs about their views of the inefficiencies in trial conduct.

The aim of this survey was to identify important inefficiencies during two key stages of the trial conduct life cycle: (i) from grant award to first participant and (ii) from first participant to reporting of final results.

## Methods

The survey was developed by the Efficient Trial Conduct subgroup of the registered CTUs work programme (http://www.ukcrc.org/research-infrastructure/clinical-trials-units/). Respondents were asked to list their top three inefficiencies from grant award to recruitment of the first participant, and their top three inefficiencies from recruitment of the first participant to publication of results. There was additional free text space for respondents to explain why they thought these were important, if they wished. The survey was simple and easy to complete, and we wanted to seek responses from a wide range of job roles within the CTUs.

The survey was constructed online using SurveyMonkey. A link to the survey was circulated in May 2013 to 45 registered CTUs (the number of units registered at that time) using the email distribution lists for quality assurance, information systems, statistics, trial managers and pharmacovigilance. An email reminder was sent to all distribution lists after two weeks. Responses were received up to 1 July 2013. Respondents were asked the name of their CTU and their job title, but all responses were otherwise anonymous. Free-text responses were coded into broad categories.

## Results

Overall, there were 43 respondents from 25 registered CTUs. Multiple responses from different respondents within the same CTU were included in the analysis: 13 units returned a single response, six submitted two responses, two submitted three responses and two units submitted five. Responses were received from units across the four nations (England, Wales, Scotland and Northern Ireland). One third of the respondents reported their job title as within trial management, and a fifth reported that they were CTU directors or in senior management (Table 1).

**Table 1:** Job titles for respondents

	*n* = 43	
Trial management (trial manager or coordinator)	14	33%
Director or senior management	8	19%
Research or programme manager	6	14%
Statistician	5	12%
Quality assurance	3	7%
IT or programmer	2	5%
Other^a^	5	12%

^a^Professor or associate professor (*n* = 2), research fellow (*n* = 2), data manager (*n* = 1)

### Responses to question: ‘*Between grant award and recruitment of the first participant, what do you think are the top three inefficiencies in trial conduct?’*

**Fig. 1:**
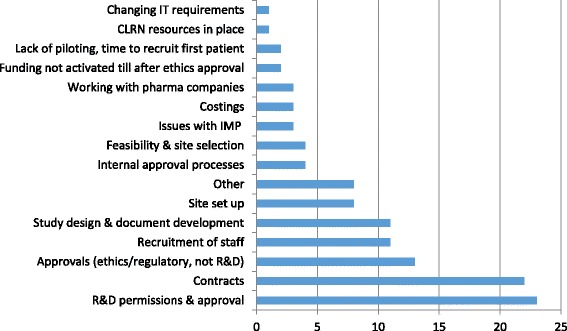
Inefficiencies between grant award and recruitment of first participant. CLRN, comprehensive local research network; IMP, investigational medicinal product; IT, information technology; R&D, research and development

Delays in obtaining research and development (R&D) permissions and approvals were reported as the top inefficiency by 23 respondents (53%); contracts by 22 (51%), and other approvals by 13 (30%) (Fig. 1). Many of those who reported R&D approvals as an inefficiency did not explain why they thought this was important. Others commented on the lack of change, and need for more consistency:

*No one believes it’s [R&D approvals] getting better – the trusts are clearly gaming it by starting and stopping the clock when they feel like it*.

*R&D’s individual requirements for what should be a standardised process for R&D approval*

The reported inefficiencies associated with contracts included all types of contract: between the funder and sponsor, between the sponsor and site or other subcontractor, and with suppliers of the investigational medicinal product.

Recruitment of staff to work on the trial was reported as an inefficiency between grant award and recruitment of the first participant by 11 (26%) respondents. Largely, this seemed to be due to delays in recruiting staff to work on the trial, although sometimes it was not clear from these comments whether this was staff in the unit or staff at sites. For some units, funders not releasing the grant until ethics approval had been secured was a significant problem contributing to delays and inefficiency:

*Issue when no core funding is in place to provide some core staff who can start projects before the trial-specific coordinators or trial managers are in post, funded by the actual grant.*

*…the need for seedcorn funding to do tasks every study requires. Clearly, seedcorn funding is better than having to subsidise the activity from another project budget or CTU support funding, but the idea that doing the pre-ethics work should be subject to a separate application is insane.*

The same number of respondents noted that study design and document development was inefficient. This was commonly noted as developing and testing the case report form (or electronic case report form), but also included agreeing the protocol. Comments included:

*Getting the investigators to decide the real detail of exactly what they are doing.*

*Design of a robust case report form with adequate PI [principal investigator] or nurse input.*

Other reported inefficiencies included selection of sites to participate in the trial, poor feasibility and piloting of the trial at sites, and lack of adequate site training. Typical comments were:

*Poor assessment of feasibility by participating sites (including potential evaluable patients).*

*Robust feasibility of deliverability.*

*Site initiation and training can be difficult as often site staff are not available or are lacking GCP [good clinical practice] training, which causes delays.*

### Responses to question: *‘From recruitment of the first patient to publication of the trial results, what do you think are the top three inefficiencies in trial conduct?’*

**Fig. 2:**
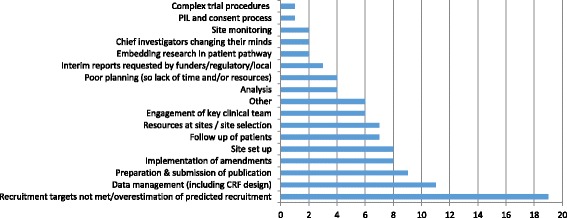
Inefficiencies between recruitment of first participant and publication of trial results. CRF, case report form; PIL, participant information leaflet

The clear front-runner as the top inefficiency for this section was ‘recruitment targets not met or overestimation of predicted recruitment’, reported by 19 (44%) respondents (Fig. 2). A common comment was that this failure to meet recruitment targets reflected overoptimistic or inaccurate estimates of recruitment at site:

*Sites wildly over-estimating suitable patient availability.*

*Failure to recruit – often because eligibility criteria are too tight and have to be widened.*

*Inaccurate estimations of likely number of eligible patients per site.*

Out-of-hours recruitment was also noted as an issue for some trials:

*… we work in emergency care trials and having 24/7 screening is paramount to success as patients can come in at any time of day. Often sites are reliant on a research nurse who is only available during office hours. We have also had a number of times when we have had to suspend sites who could not recruit patients when no research nurse was in place (moved jobs) and it may have taken 8–9 months to replace the nurse.*

Data collection, including case report form design, was reported as an important inefficiency by 11 (26%) respondents, and preparation and submission for publication by 9 (21%). Many of the issues noted with data management related to delays in resolving data queries with sites:

*Timely data flow (related to site staff availability or turnover).*

*Final clean dataset for analysis: data queries are not resolved in a timely manner from sites.*

For preparation and submission for publication, several respondents commented that delays were due to this often taking place after the end of the grant, when key staff might have left the project:

*Time and resources allocated to produce publication (which is after grant end).*

One comment was that negative findings might not get published at all:

*Publication bias – negative findings not published.*

Inefficiencies in data management may contribute to delays or inefficiencies in analysis and preparing results for publication. One respondent reported how recognising this and improving the efficiency of data management improved efficiency in preparing the final report:

*Obviously, if you fail to plan ahead – you delay the analysis and give less time for the report writing. Glad to be blaming ourselves rather than someone else for this one. We’ve realised that we can cut down the time for data cleaning after follow-up is complete – to allow speedy transfer of data to analysts – if we step up the process of query resolution from 6 months before the last participant’s last visit and get statisticians, health economists and DM [data manager] looking at blinded sample data to anticipate where the problems will be early. We’ve put a lot of work into trying to make this bit of the trial more efficient recently. The CI [chief investigator] sometimes gets in the way, but mostly they’re pleased and impressed that we’re thinking ahead like that.*

Site set-up and implementing ethics approval amendments were reported as a top inefficiency by a fifth of respondents (8, 19%). For ethics amendments, the issues included time taken to secure amendments, and delays in R&D to implement the amendment at sites.

Site selection and resources at sites were noted as inefficiencies by several respondents:

*Clinicians not having time or adequate support.*

*Lack of research nurse time to identify patients.*

Poor engagement of the key clinical team, both chief investigators and at sites, was also reported as an inefficiency:

*Minimal communication between clinicians, trial managers and statisticians during trial conduct.*

*Chief investigators not allocating sufficient time and focus on trial.*

*Lost motivation from clinical staff.*

Various aspects of project planning were also reported as inefficiencies, including planning the patient pathway, study monitoring and end-of-study planning:

*Ramifications of poor planning at grant application stage can result in drug supply issues, increased costs or interruptions to IMP [investigational medicinal product] supply, changes to eCRF [electronic case report form] system after study start, under-recruitment, etc.*

*Insufficient resource allocated to trial management or marketing of the trial.*

## Discussion

It is estimated that 85% of research is wasted [3]. Factors contributing to research waste include conducting studies that address questions of low importance to patients and clinicians [4], that are designed without reference to a systematic review of the evidence [4], that fail to take adequate steps to reduce bias [5], and that fail to report, or that inadequately report, their results [6]. Improving the efficiency of trial conduct is clearly an important strategy for reducing such waste [1, 7]. Respondents to our survey represented a wide range of roles and CTUs, although the response rate was lower than expected (owing to a problem with the email distribution list, subsequently corrected but not in time for our response rate). The top reported inefficiencies present no surprises: securing necessary approvals and permission [8–10], poor recruitment [11, 12] and data management [13] are all well recognised as challenges to efficient trial conduct. Many of the other issues identified relate to project planning, such as doing appropriate pilot and feasibility assessment, and selecting and maintaining good sites (good at both recruitment and data collection), setting realistic recruitment targets and developing high-quality documents and data collection tools. This emphasises the importance of having sufficient time and expertise in the early planning of a trial [13, 14], and offers insight into a range of problems facing units conducting multicentre trials. In view of the importance of early planning for efficient trial conduct, it would be useful to have early input, ideally from the stage of preparing the grant application, from an experienced trial manager.

Several respondents noted the potential for recent improvements in the process of securing research ethics approval for multicentre studies (https://www.myresearchproject.org.uk/SignIn.aspx) and for obtaining local health service approval at each site (http://www.ukcrc.org/regulation-governance/streamlining-rd-permissions/national-systems-for-rd-permissions/) to improve efficiency and reduce delays. Nevertheless, gaining approvals remained the top reported inefficiency. In England, in 2016, the Health Research Authority completed the introduction of a new process called Health Research Authority Approval, which streamlines within a single process the assessment of governance and legal compliance (by dedicated Health Research Authority staff), with review by an independent research ethics committee (http://www.hra.nhs.uk/about-the-hra/our-plans-and-projects/assessment-approval/). This appears to have reduced time to approval, but merits evaluation to assess impact on overall efficiency. Contract negotiations are another issue often outside the direct influence of an individual unit or research team. Similarly beyond direct control by the unit, working with external suppliers such as pharmaceutical companies and issues around the supply of investigational medicinal product were reported as inefficiencies by a few respondents. Clearly, these are only issues for units that conduct clinical trials of investigational medicinal products, which not all do, as some only conduct trials of complex interventions or medical devices.

Meeting recruitment targets is clearly a key challenge for all trials. Responses in this survey indicate the wide range of factors that can contribute to poor recruitment, but also offer some insight into their potential solutions. For example, recruitment targets should be realistic not only for the study overall but also for individual sites. Realistic targets for sites should be based on information from those sites, which means collecting appropriate data on the target population and assessing the patient pathway. Meeting recruitment targets also depends on selecting the right sites; hence, making better-informed decisions about site selection, checking for competing trials at sites, and strong engagement by the local investigators will all improve efficiency in recruitment. Using simple questionnaires to gather relevant information from potential sites may improve selection of sites. Prompt recognition of problems with recruitment will facilitate rapid remedial action. Lack of engagement from the study’s chief investigator, and poor communication between the chief investigator and the project team, were also noted as contributing to inefficiency.

Recruitment and training of staff were reported as issues in inefficiency of trial conduct. For some responses it was unclear whether the problem was recruitment at the trials unit or at sites. However, it was clear that delays in recruiting appropriate staff to work on the project once the grant was awarded could be a major problem. Delays at this early stage can have considerable impact throughout the study, as delays may be cumulative and once a study falls behind target it can be difficult to catch up.

Since our survey was conducted, several factors may have contributed to potential improvement in efficient trial conduct. As discussed, the approval process has been changed and streamlined. The importance of methodological research to increase our knowledge about how to improve the efficiency and quality of trial conduct, in particular how to improve strategies for trial management, is now more widely recognised. Initiatives to raise awareness of and to facilitate such research include the SWAT (study within a trial) programme [15] and Trial Forge, which aims to aims to increase the evidence base for trial decision making [2]. The biennial International Clinical Trials Methodology Conference hosted by the Network of Hubs for Trials Methodology Research has become well established, and provides a forum for those interested in improving efficient trial conduct to network, share experiences and present their research.

Our survey addressed inefficiencies in the conduct of individual trials, which contribute to waste in research [7]. There are also broader issues with inefficiencies in trial conduct that contribute to research waste, however [16], for example how studies are selected for funding [4], inaccessibility of full information about published studies [17] and failure to report about half of all clinical trials [3] (http://www.alltrials.net/). Advocacy for transparency in clinical trials is accelerating, and is supported by hundreds of institutions, including the UKCRC registered CTUs network (http://c.ymcdn.com/sites/www.ukcrc-ctu.org.uk/resource/resmgr/ukcrc_response_eu_regs_summa.pdf).

## Conclusions

Recommendations for improving the efficiency of trial conduct for multicentre trials include:

- Applying leverage for further reducing unnecessary bureaucracy in approvals and contracting
- Improving training for site staff, for example by developing ways for CTUs to share knowledge about sites and work together to provide site training
- Improving the working relationships between chief investigators and CTUs, for example by developing guidance on their respective roles and responsibilities, including the importance of realistic recruitment targets and feasibility for efficient planning and conduct
- Sharing good practice across units and developing training across the network of units
- Encouraging funders to release sufficient funding to allow prompt recruitment of trial staff
- As we need better information about how to improve efficient trial conduct, CTUs should encourage research whenever possible to improve our knowledge about how to improve the efficiency and quality of trial conduct

## Abbreviations

CTU: Clinical Trials Unit
R & D: Research and development
SWAT: Study within a trial
UKCRC: UK Clinical Research Collaboration
